# Small RNA mediated regulation of seed germination

**DOI:** 10.3389/fpls.2015.00828

**Published:** 2015-10-13

**Authors:** Shabari Sarkar Das, Prakash Karmakar, Asis Kumar Nandi, Neeti Sanan-Mishra

**Affiliations:** ^1^Plant Molecular Biology Group, International Centre for Genetic Engineering and Biotechnology, New Delhi, India; ^2^Department of Botany and Forestry, Vidyasagar University, Midnapore, West Bengal, India

**Keywords:** seed germination, seed, small RNA, miRNA, stress response, seed dormancy

## Abstract

Mature seeds of most of the higher plants harbor dormant embryos and go through the complex process of germination under favorable environmental conditions. The germination process involves dynamic physiological, cellular and metabolic events that are controlled by the interplay of several gene products and different phytohormones. The small non-coding RNAs comprise key regulatory modules in the process of seed dormancy and germination. Recent studies have implicated the small RNAs in plant growth in correlation with various plant physiological processes including hormone signaling and stress response. In this review we provide a brief overview of the regulation of seed germination or dormancy while emphasizing on the current understanding of the role of small RNAs in this regard. We have also highlighted specific examples of stress responsive small RNAs in seed germination and discussed their future potential.

## Introduction

**FIGURE 1:**
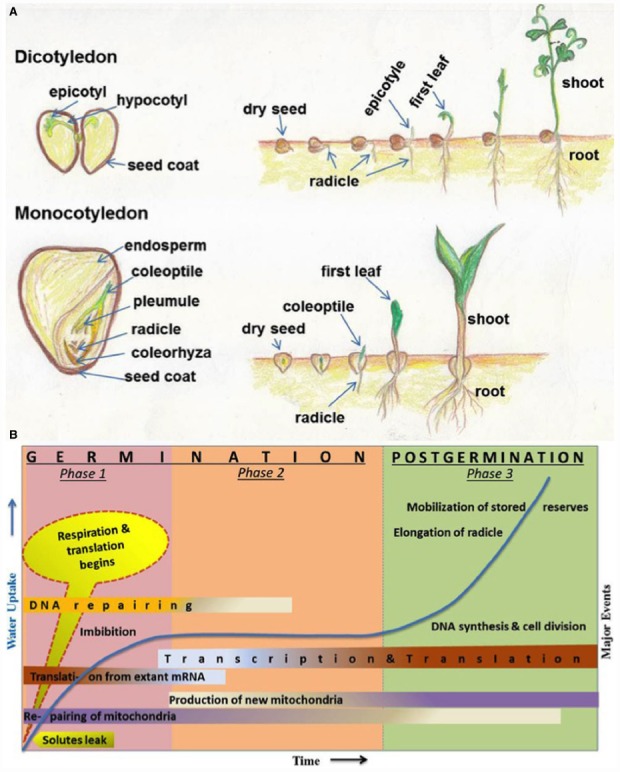
**(A)** Schematic representation (hand drawn by SS) of different parts of seeds and seed germination stages. Seeds and germination stages of dicotyledonous (chickpea) and monocotyledonous (maize) plants have been shown in upper and lower panels, respectively. **(B)** Major events associated with seed germination and post-germinative growth phases. Germination stages are represented by phase 1 and phase 2; postgermination events includes phase 3. The time (x-axis) for events varies from several hours to many weeks, depending on different plant species and germination conditions. Uptake of water and related increase in biomass is indicated in y-axis and shown in line graph during three phases. Some events (such as DNA repairing, transcription, translation, and mitochondria production etc.) are spread over more than one phases and indicated with shaded color; dark colors indicate more activity and light colors indicate less activity. This figure has been reproduced with modification, after written permission of the corresponding author (Prof. J. D. Bewley) and the original publisher, American Society of Plant Biologists (ASPB).

The seeds of higher plants contain the dormant embryos, as miniature new plants, along with adequate food reserves to sustain the growing seedlings until they establish themselves as self-sufficient, autotrophic organisms (Figure 1A). Germination is one of the most important physiological process of a seed which begins with the uptake of water by the quiescent dry seed and is completed when a part of the embryo, usually a radical oozes out of the seed coat (Bewley, 1997). Seed dormancy is regarded as the temporary failure or block of a viable seed to complete germination under seemingly unfavorable conditions and is an adaptive feature for optimizing the timing of germination (Bewley, 1997). The dynamic process of seed germination is triphasic (Figure 1B) and involves a complex coordination of many physiological, cellular and metabolic events (Bewley, 1997; Weitbrecht et al., 2011). Phase-I includes rapid leakage of solutes which paves the way for respiration and protein synthesis. Phase-II represents a plateau stage where new mRNAs and proteins are synthesized. There is also an accumulation of the mitochondrion to support the energy requirements at this stage. During phase-III, radicle cells elongate and divide. This is also the stage of rapid DNA synthesis and replication together with the mobilization of stored reserves (Bewley, 1997).

Various environmental factors such as light, temperature, moisture, oxygen, soil, humidity, stress etc and some physiological factors such as viability of seeds, thickness of seed coat, dormancy period etc also play vital role in seed germination stages (Martin et al., 2010; Weitbrecht et al., 2011). Several studies have implicated that the interactions between different phytohormones such as abscisic acid (ABA), gibberellins (GAs), ethylene, brassinosteroids (BRs), auxin, and cytokinins (CKs) play a key role in regulating the interconnected molecular processes that control dormancy release and activation of the stages of seed germination (Liu et al., 2007; Finkelstein et al., 2008). The activity of plant hormones needs to be precisely regulated, since some phytohormones exert crucial but contrasting influence on the process. ABA is a positive regulator of dormancy and its maintenance, while it is a negative regulator of germination (Finkelstein et al., 2008). The absence of, or insensitivity to ABA during seed development results in the production of viviparous or precociously germinating seeds as exemplified by maize *viviparous* (*vp*), tomato *sitiens* (*sit*), and *Arabidopsis ABA-deficient* (*aba*), and *ABA-insensitive (abi*) mutants (Finkelstein et al., 2008). GA releases dormancy and its role is analogous to that of Ethylene and BR in promoting germination by counteracting ABA effects. Recently, the crosstalk between ABA and auxin has also been highlighted (Finkelstein et al., 2008).

The discovery of small non-coding RNAs (of 19–24 nucleotides length) has added a new dimension to the understanding of the regulation of cellular environment (Bartel, 2004; Axtell et al., 2007). They have been shown to play diverse roles in growth, development, morphogenesis and stress responses of both plants and animals (Chen, 2012; Kamthan et al., 2015). The functional small RNAs are produced from double stranded RNA precursors through the activity of RNA-dependent RNA Polymerase (RDR), DICER-like (DCL), and ARGONAUTE (AGO) proteins (Allen et al., 2005; Mallory et al., 2008; Axtell, 2013). There are two major classes of small non-coding RNAs—short interfering RNAs (siRNAs) and microRNAs (miRNAs) that negatively regulate their target genes by binding to the complementary sequences. At the transcriptional level, the small RNAs may be involved in chromatin remodeling (Huettel et al., 2007; Pontier et al., 2012; Xie and Yu, 2015) while at the post-transcriptional level, depending upon the nature of homology they can bring about the cleavage of the target mRNA (Rajagopalan et al., 2006; Vaucheret, 2006) or block their translation (Poethig et al., 2006; Vaucheret, 2006; Bartel, 2009). The biosynthesis and function of many of these small RNA genes are also regulated by different plant hormones and environmental stress (Mallory et al., 2005; Reyes and Chua, 2007; Sunkar et al., 2007; Shukla et al., 2008; Martin et al., 2010; Khraiwesh et al., 2011; Sanan-Mishra et al., 2013).

## Biogenesis of miRNA and ta-siRNA

miRNA biogenesis is a multistep process that is mainly resistricted to the nucleus in plants. Briefly, the miRNA gene is transcribed into a capped and poly-adenylated primary miRNA (pri-miRNA) by enzyme RNA polymerase II (Chen, 2012; Axtell, 2013). The pri-miRNA is processed to precursor miRNA (pre-miRNA), of around 70–100 nt long, by DCL protein (Axtell, 2013). The pre-miRNA is further processed to form miRNA and miRNA* duplex by the activity of DCL protein. The duplex is then methylated at the 2′OH of the 3′nucleotide end by HEN1 (Allen et al., 2005; Chen, 2009) and transported to the cytoplasm.

One strand of the duplex is loaded into RISC (RNA-induced silencing complex) containing AGO1. The strand selection widely depends on the relative stability of the two ends of the duplex (Axtell, 2013). It is observed that generally the strand whose 5′ end is comparatively loose, gets incorporated into the RISC (Allen et al., 2005; Axtell, 2013). The RISC complex containing the miRNA identifies its target transcripts based on perfect or nearly perfect sequence complementarity. In plants the stringency of target recognition is very high and the target transcripts are normally cleaved, however, the central mismatches in the miRNA:mRNA pair direct the inhibition of translation (Allen et al., 2005; Axtell, 2013).

Recently, the ta-siRNA (trans-acting small interferring RNAs) have also been implicated in plant development thereby attracting major research interest for many plant biologists (Nogueira et al., 2006; Axtell, 2013). ta-siRNAs are generated from TAS (Trans-Acting SiRNA locus) gene derived non-coding transcripts through specific miRNA guided cleavage. The cleaved precursors of ta-siRNAs are bounded and stabilized by SUPPRESSOR of GENE SILENCING3 (SGS3) and further synthesized into double stranded RNAs by RDR6 (Chen, 2009; Allen and Howell, 2010; Axtell, 2013). The double stranded RNAs are cleaved several times by DCL4 from the miRNA mediated cleavage sites, so that 21-nt long phased ta-siRNAs are produced. Similar to miRNAs, the ta-siRNAs are incorporated into RISCs, where they cleave the target mRNAs or repress translation (Allen et al., 2005; Allen and Howell, 2010). There are four families of TAS gene in *Arabidopsis*, namely *TAS1*, *TAS2*, *TAS3*, *TAS4* (Rajagopalan et al., 2006; Allen and Howell, 2010)*.* For the initial processing *TAS1* and *2* require miR173 whereas *TAS3* and *TAS4* require miR390 and miR828, respectively for initial processing (Chen, 2009; Allen and Howell, 2010; Axtell, 2013).

## The Role of miRNAs and ta-siRNAs in Plant Growth and Development

The miRNAs constitute a major class that play important and diverse roles in regulation of various aspects of plant development (Sanan-Mishra and Mukherjee, 2007; Chen, 2012; Sharma et al., 2015). The classical examples include regulation of *CUC1/CUC2* and *NAC1* transcripts by miR164 to affect reproductive and root development (Guo et al., 2005); determination of abaxial/adaxial leaf polarity and root development by miR166/165 mediated control of *Class III HD-ZIP* transcription factor mRNAs (Chen, 2012; Barik et al., 2014; Singh et al., 2014) and the regulation of flower development in *Arabidopsis thaliana* by miR172 targeted AP2 and other mRNAs (Wollmann et al., 2010). The function of miRs has been shown to be affected by hormones and stress responses (Mallory et al., 2005; Liu et al., 2007; Reyes and Chua, 2007)

The miRNA mediated, ta-siRNA production is also significantly altered in drought, salinity and hypoxia stresses, besides their regulation by auxin and other hormones (Moldovan et al., 2009; Matsui et al., 2014). This is evident by *TAS3* derived ta-siR-ARF that target different *AUXIN RESPONSE FACTOR2, 3 and 4 (ARF2,3,4)* and regulate various aspects of plant development such as vegetative to reproductive phase changes, leaf polarity and lateral root development in *Arabidopsis* (Peragine et al., 2004; Chitwood et al., 2007; Allen and Howell, 2010; Marin et al., 2010). Mutations in ta-siRNA biogenesis pathway lead to the upregulation of target mRNAs and affect the aforesaid aspects of plant development. The rice and maize ta-siRNA biogenesis mutants have been shown to have severely affected shoot and leaf development (Itoh et al., 2006; Nogueira et al., 2006; Nagasaki et al., 2007; Douglas et al., 2010). DCL4 is suggested to redundantly regulate processing of some miRNAs, besides role in ta-siRNA biogenesis (Rajagopalan et al., 2006). ABA signaling is shown to be, at least partially, affect RDR6 accumulation (Zhang et al., 2013). Although ta-siRNA has not directly been implicated in seed germination, their cross talk with miRNA and hormone signaling in feed-back loops (Marin et al., 2010; Chen, 2012) as well as role in seed development (Zhang et al., 2013) indicate their potential function in seed maturation and germination. This remains to be an interesting area to be explored in the complex process of seed germination.

## Molecular Network of Small RNAs in Seed Germination and Dormancy

Throughout the life cycle of an angiosperm plant, there are two major developmental phase transitional periods. One is germination (from seed to seedling stage; Huang et al., 2013) and another is flowering (from vegetative to reproductive stage; Wu et al., 2009). Recent studies indicate that genes, regulating phase transition to flowering are also involved in transition from dormancy to germination (Huang et al., 2013). The genes that regulate cellular phase transitions from embryo to seedling growth also play important role in the process. In addition, phytohormones and environmental factors affect expression of seed germination (Liu et al., 2007; Finkelstein et al., 2008). Recently, a role for small RNAs has been indicated in this process by characterizing the mutants of small RNA biogenesis pathway genes, such as *DCL1*, *HYL1*, *HEN1*, and *AGO1* that display severe defects in embryogenesis and seed development (Willmann et al., 2011). This can be illustrated by the *dcl1* mutant, which shows early seed maturation phenotype than the normal wild type seeds. The positive regulators of *DCL1* gene are leafy cotyledon (*LEC*) genes like *LEC2* and *FUS3*. Whereas the negative regulators or repressors of early embryo maturation are *ASIL1*, *ASIL2*, and *HDA6/SIL1* (Willmann et al., 2011).

Different miRNAs like are miR160, miR159, miR417, miR395, miR402, mir165/166, miR164, miR167, miR156, miR172, and miR158 (Table 1) are known to control both the activators and repressors of seed germination and dormancy (Jung and Kang, 2007; Liu et al., 2007; Reyes and Chua, 2007; Kim et al., 2010a,b; Martin et al., 2010; Huang et al., 2013). Increased level of miR156 and reduced level of SPLs and miR172 (Table 1) in the mature embryo could down regulate the developmental transition and keep seeds in dormant stages (Martin et al., 2010; Huang et al., 2013). The imbibition step itself has been shown to differentially down-regulate twelve miRNA families, miR156, miR159, miR164, miR166, miR167, miR168, miR169, miR172, miR319, miR393, miR394, and miR397; while four families, miR398, miR408, miR528, and miR529 were up-regulated during the seed germination (Li et al., 2013). Interestingly, miR156 and miR157 have also been implicated in vegetative to reproductive phase change (Wu et al., 2009), indicating their functional diversification.

**TABLE 1:** **List of miRNAs that are involved in seed germination and dormancy**.

**miRNAs**	**Targets of miRNA**	**Target regulation**		**Seed germination related function**	**References**
		**Up-regulation**	**Down-regulation**	
miR395	ATP Sulfurylases (*APS1*,* APS3*, *APS4*); Sulfate transporter (*SULTR2:1*)	*APS1*,* APS4*,* SULTR2:1* by miR395c	*APS1*, *APS4*, *SULTR2:1* by miR395c and *APS3 by miR395e*	Regulatory effects on seed germination under salt and dehydration stress	Kim et al. (2010b)
miR402	DEMETER-LIKE protein 3* (DML3*)		↓	Regulatory effect on seed germination and seedling growth under salt, dehydration and cold stress	Kim et al. (2010a)
miR417	Unknown	Unknown	Unknown	Plays a role as a negative regulator of seed germination in Arabidopsis thaliana under salt stress condition.	Jung and Kang (2007)
miR160	*ARF10*,* ARF16*, *ARF17*		↓	*ARF10* mutant show up regulation of ABA responsive genes during germination	Liu et al. (2007)
miR159	*MYB33*,* MYB65*,* MYB101*	*MYB33*,* MYB101*		*MYB33* and *MYB101* are the positive regulators of ABA signaling during seed dormancy and germination	Reyes and Chua (2007)
miR165/166	*PHB*, *PHV*, *REV* etc.	Unknown	Unknown	Maintain the auxin signal during seed development and maturation.	Huang et al. (2013)
miR164	*NAC1*, *CUC1/CUC2*	Unknown	Unknown	Maintain the auxin signal during seed development and maturation.	Huang et al. (2013)
miR167	*ARF6*, *ARF8*	Unknown	Unknown	Maintain the auxin signal during seed development and maturation.	
miR158	Unknown	Unknown	Unknown	Seed development and maturation.	
miR156	*SPL* 3, 4, 5		↓	Seed development and maturation.	Huang et al. (2013); Li et al. (2013)
miR172	*AP2*	↑		Seed development and maturation.	

*The first five miRNAs in the gray shaded region of the table are also involved in mediating the stress response signals during germination*.

The complex regulatory cross-talk between the hormones and the small RNAs, was evident by the identification of two ABA supersensitive mutants for germination viz. *absg1* and *absg2* as the alleles of *dcl1* and *hen1*. The *absg1* and *absg2* mutants show up regulation of the expression of ABA responsive genes (Zhang et al., 2008). An important role for miR159 has been demonstrated in regulating the dynamic seed germination procedure by modulating GA and ABA hormone signaling (Table 1). The expression of miR159 is controlled by both GA and ABA (Martin et al., 2010). The GAMYB proteins act as the positive regulators, whereas DELLA proteins act as the negative regulators of the GA signaling cascade (Peng and Harberd, 2002; Finkelstein et al., 2008; Weitbrecht et al., 2011). The GAMYB mRNAs are regulated by miR159 during floral development, fertility and seed germination (Reyes and Chua, 2007). Recently, it was shown that alurone vacuolation, a GA-mediated (GAMYB protein) programmed cell death (PCD) process in alurone is required for seed germination (Peng and Harberd, 2002; Finkelstein et al., 2008; Alonso-Peral et al., 2010). The miR159 also regulates transcription factors MYB33 and MYB101, which are the positive regulators of ABA signaling during seed dormancy and germination (Reyes and Chua, 2007; Martin et al., 2010). miR159 expression is upregulated in *rdr2* and *dcl2 dcl3 dcl4* triple mutants. Interestingly, RDR2, DCL2, DCL3, DCL4 are the essential factors in case of siRNA biogenesis, especially heterochromatic siRNA biogenesis pathway (Allen and Howell, 2010; Axtell, 2013). This suggests that different kinds of small RNAs, besides miRNAs, could essentially play significant role in seed germination and dormancy.

The role of phytohormone Auxin in seed germination, became evident when Liu et al. (2007) showed that miR160 mediated down regulation of ARF10 plays crucial roles in seed germination (Table 1; Liu et al., 2007). ARFs are transcription factors involved in auxin signaling pathway during many plant growth and developmental stages. The miR160 also appears to be the converging point of Auxin and ABA mediated cross-talk during seed germination, since mutation in ARF10 results in developmental defects and overexpression of ABA responsive genes (Liu et al., 2007). Similarly, it was shown that over expression of miR160 caused hyposensitivity to ABA during germination (Liu et al., 2007). Auxin homeostasis is vital for embryo development and is mediated by the action of miR165/166, miR167, miR164, miR158, and miR160 (Martin et al., 2010). This suggests an important role for the miRNAs in mediating suitable auxin signaling during embryo and seed development (Table 1). Thus, it could be concluded that these miRNAs play important roles in maintaining dormancy and breaking of dormancy to promote embryo into seedling stage through seed germination (Martin et al., 2010; Huang et al., 2013; Zhang et al., 2013).

Gaseous hormone ethylene promotes seed germination through interaction with ABA signaling (Finkelstein et al., 2008) The two mutants namely *ethylene resistant1 (etr1)* and *ethylene insensitive2 (ein2)* or, *enhanced response to aba3 (era3)* show upregulation of ABA responsive genes and delay in seed germination (Finkelstein et al., 2008).Whereas wild type seeds treated with ethylene precursor ACC (1-aminocyclopropane -1-carboxylic acid) show downregulation of ABA response factors (Finkelstein et al., 2008). Again, *etr1-2* mutant show the over accumulation of GA content, which could be a compensation to over accumulation of ABA (Finkelstein et al., 2008). Since miR160 and miR159 both have regulatory effects on ABA and GA, and ethylene has a cross talk with ABA and GA, therefore, it is hypothesized that these miRNAs may have direct or indirect control over ethylene mediated regulation during seed germination and dormancy.

Plant steroid hormone BRs that mainly effect stem elongation and leaf unfurling also effect seed germination. The mutants for BR biosynthetic and signaling pathway are sensitive to ABA leading to decrease in the germination potential (Finkelstein et al., 2008). The possibility of a cross talk between BR and ABA signaling cannot be ruled out in the activation of the miR160 regulatory pathway in seed germination (Liu et al., 2007). Also, BRs induce the expression of distinct *EXPANSIN (EXP)* family members, which are cell wall loosening proteins that can indirectly influence seed germination (Bewley, 1997).

Parallel studies have shown that the small RNA biogenesis pathway mutants, that show high expression of ABA, are highly sensitive to salt and osmotic stresses (Zhang et al., 2008), thereby indicating the overlap with the environmental cues. Under abiotic stress conditions, miR395 (Table 1) acts both as a positive and negative regulator of seed germination (Kim et al., 2010b). miR395 has six family members in *Arabidopsis* genome, that target the proteins APS1, APS3, APS4, and SULTR, involved in sulfate assimilation and transport. It was shown that miR395e that differs from miR395c in a single nucleotide cannot target APS1 and APS4. These miRNAs have different effects on the seed germination of *Arabidopsis* under high salt or dehydration stress conditions (Kim et al., 2010b). Over expression of miR395c reduces the germination potential under high salt or dehydration stress condition; whereas over expression of miR395e enhances the germination potential under the same stress condition in *Arabidopsis thaliana* (Kim et al., 2010b). Similarly over expression of miR402 (Table 1) enhances the seed germination potential in *Arabidopsis* under salt, dehydration and cold stress conditions (Kim et al., 2010a). miR402 downregulates its target gene *DML3 (DEMETER-LIKE protein3)*, which is involved in DNA demethylation, an epigenetic regulatory process of plants in various stress conditions (Kim et al., 2010a). miR417 (Table 1) also exhibits a negative regulation over seed germination under salt stress condition (Jung and Kang, 2007). However, its mechanism of molecular action is not yet clear.

## Conclusion and Future Perspective

Agriculture exclusively depends on growing crops; so the success of cultivation as well as productivity largely depends on seed viability, seed germination and efficiency of seed development. Small RNAs play critical roles in regulation of gene expression in developing and germinating seeds (Kamthan et al., 2015). In this review we describe that specific small RNAs, mainly miRNAs regulated nodes, play crucial roles in regulating seed germination in response to different phyto-hormones and abiotic stresses. But the mechanism of action and the interconnection of the various signaling cascades with their regulatory networks remain largely unknown till date. Thus, functional analysis of small RNAs expressed in seeds or during germination process will provide useful information for seed biology. Future studies are required to unravel the molecular details of small RNAs regulated pathways in seed germination and viability maintenance, and their association with the stress responses and hormonal signals, especially in crop plants. Expression and functional analysis using transgenic approach, proteomic analysis and the use of different bioinformatics tools could also help to throw light on this issue.

### Conflict of Interest Statement

The authors declare that the research was conducted in the absence of any commercial or financial relationships that could be construed as a potential conflict of interest.
